# The Role of RNA and DNA Aptamers in Glioblastoma Diagnosis and Therapy: A Systematic Review of the Literature

**DOI:** 10.3390/cancers12082173

**Published:** 2020-08-05

**Authors:** Silvia Nuzzo, Valentina Brancato, Alessandra Affinito, Marco Salvatore, Carlo Cavaliere, Gerolama Condorelli

**Affiliations:** 1 IRCCS SDN (Istituto di Ricovero e Cura a Carattere Scientifico, SYNLAB istituto di Diagnostica Nucleare), 80131 Naples, Italy; silvia.nuzzo@synlab.it (S.N.); direzionescientifica@sdn-napoli.it (M.S.); carlo.cavaliere@synlab.it (C.C.); 2 Percuros B.V., 2333 CL Leiden, The Netherlands; a.affinito@percuros.com; 3 Department of Molecular Medicine and Medical Biotechnology, “Federico II” University of Naples, Via Tommaso de Amicis 95, 80131 Naples, Italy; 4 IRCCS Neuromed–Istituto Neurologico Mediterraneo Pozzilli, 18–IT-86077 Pozzilli, Italy

**Keywords:** aptamer, nucleic acid, glioblastoma, diagnosis, therapy

## Abstract

Glioblastoma (GBM) is the most lethal primary brain tumor of the central nervous system in adults. Despite advances in surgical and medical neuro-oncology, the median survival is about 15 months. For this reason, initial diagnosis, prognosis, and targeted therapy of GBM represent very attractive areas of study. Aptamers are short three-dimensional structures of single-stranded nucleic acids (RNA or DNA), identified by an in vitro process, named systematic evolution of ligands by exponential enrichment (SELEX), starting from a partially random oligonucleotide library. They bind to a molecular target with high affinity and specificity and can be easily modified to optimize binding affinity and selectivity. Thanks to their properties (low immunogenicity and toxicity, long stability, and low production variability), a large number of aptamers have been selected against GBM biomarkers and provide specific imaging agents and therapeutics to improve the diagnosis and treatment of GBM. However, the use of aptamers in GBM diagnosis and treatment still represents an underdeveloped topic, mainly due to limited literature in the research world. On these bases, we performed a systematic review aimed at summarizing current knowledge on the new promising DNA and RNA aptamer-based molecules for GBM diagnosis and treatment. Thirty-eight studies from 2000 were included and investigated. Seventeen involved the use of aptamers for GBM diagnosis and 21 for GBM therapy. Our findings showed that a number of DNA and RNA aptamers are promising diagnostic and therapeutic tools for GBM management.

## 1. Introduction

Glioblastoma (GBM) is classified by the World Health Organization as grade IV astrocytoma and is the most common and fatal primary brain tumor of the central nervous system (CNS) in adults [1,2]. Despite advances in surgical and medical neuro-oncology, median survival is only 15 months after the first diagnosis and with standard surgery and chemoradiation [3,4,5,6,7]. For this reason, initial diagnosis and targeted therapy of GBM represent very attractive areas of study.

Currently, diagnostic methods are based on initial neurological examination and imaging tests (magnetic resonance imaging, MRI, and computerized tomography, CT) to evidence brain abnormality. Nevertheless, there are many limitations in standard GBM diagnostic methods: in fact, distinguishing glioma grades or discriminating GBM from other intracranial mass lesions remain very difficult or even impossible by conventional techniques [8]. Moreover, often only surgical biopsy can definitely confirm the diagnosis. In addition to these factors, the fact that GBM symptoms overlap with those of other brain disorders makes diagnosis even more difficult, with GBM in certain cases being misdiagnosed as other CNS pathologies (sinusitis, tension headache, myasthenia gravis, or demyelinating disease) [9].

After initial diagnosis, current clinical treatment for GBM requires a multidisciplinary approach, consisting in initial tumor resection followed by concurrent radiotherapy and chemotherapy. However, despite maximal safe surgical resection and multimodality therapy, about 70% of these tumors invariably recur, with standards of care at recurrence far less well defined than in the newly diagnosed setting [7,10,11]. Radiotherapy and chemotherapy in GBM are affected by de novo or acquired resistance, reasons why surgery and temozolomide (TMZ) treatment before or during standard care therapy can increase patient survival by only around 15 months. Therefore, novel drugs able to inhibit GBM cell growth and not subjected to the side effects of the current therapies are needed [6].

Given the above-mentioned limitations and the lack of standardization related to GBM diagnosis and treatment, the need to develop new diagnostic and therapeutic strategies for GBM diagnosis and treatment, also involving targeted imaging and drug delivery platforms, has led to the investigation of aptamers. Indeed, these molecules possess many features making them ideal novel imaging and therapeutic agents for diagnosis and treatment of GBM [12].

To date, nucleic acid aptamers have attracted growing interest as biosensors and diagnostic and therapeutic elements for tumor imaging due to the following properties: (i) chemical synthesis allowing for low batch variation, (ii) an ability to recognize targets with high specificity and affinity, (iii) a stability at high or low temperatures and critical pH values, and (iv) a small size that allows good tissue penetration and rapid clearance [13,14]. Further, aptamers can be internalized into cells to deliver payloads [15]. Moreover, very recent studies on aptamers demonstrate a capacity to cross the blood brain barrier (BBB), considered the major obstacle for innovative GBM approaches [16]. Thanks to these attractive characteristics, these innovative molecules have been studied so far to improve the quality and sensitivity of imaging techniques for diagnostic purposes [17] and to explore new therapeutic approaches for GBM management [18].

Concerning the use of aptamers in GBM therapy, various aptamers have been tested as therapeutic agents. Several studies investigated aptamer-based drugs that bind to and inhibit specific proteins, while others were on aptamers as systems for drug delivery specifically to the tumor cells. However, despite their promise, the use of aptamers in GBM diagnosis and treatment still represents an underdeveloped topic, mainly due to limited literature in the research world. In this scenario, the aim of this systematic review is to collect and summarize the current state of the art on the role of aptamers in GBM, focusing on their use in diagnostic and therapeutic applications.

## 2. Materials and Methods

### 2.1. Search Strategy and Selection Criteria

A systematic search for all published studies concerning the application of aptamers for GBM management was conducted. The most relevant scientific electronic databases (PubMed, Cochrane Library, MEDLINE, ScienceDirect, Google Scholar) were comprehensively explored and used to build the search. Only studies published since 2000 were selected. The search strategy included the key terms listed in S1.

The literature search was restricted to English language publications. Two reviewers, after having independently screened identified titles and abstracts, assessed the full text of the original articles involving aptamers used as probe or therapeutic tools for GBM management. For articles meeting these criteria with full text available, the following further selection criteria were used: articles were excluded if they also involved any type of brain tumor other than GBM and if they were off-topic after investigating the full text. Moreover, we only included articles demonstrating the utility of aptamers for human applications. The entire flow and results of the literature research were finally checked by a third researcher.

### 2.2. Data Extraction and Collection

After the above-mentioned selection procedure, selected articles that met the inclusion criteria were analyzed by two reviewers, and data useful for conducting the systematic review were extracted and collected in a pre-designed sheet. Extracted data included the following: study characteristics (first author name, year of publication, and method of study, namely in vivo and/or in vitro), name of the aptamer investigated, nucleic acid sequence, aptamer target, diagnostic or therapeutic application depending on if the study used aptamers for GBM diagnosis or therapy, and dissociation constant (Kd).

### 2.3. Planning and Conducting the Review

The articles were classified according to the purpose they had, namely if they concerned application for GBM diagnosis or therapy. This systematic review was conducted in accordance with the Preferred Reporting Items for Systematic Reviews and Meta-Analyses (PRISMA) statement (See S2 for PRISMA Checklist).

## 3. Results

### 3.1. Study Selection

**Figure 1:**
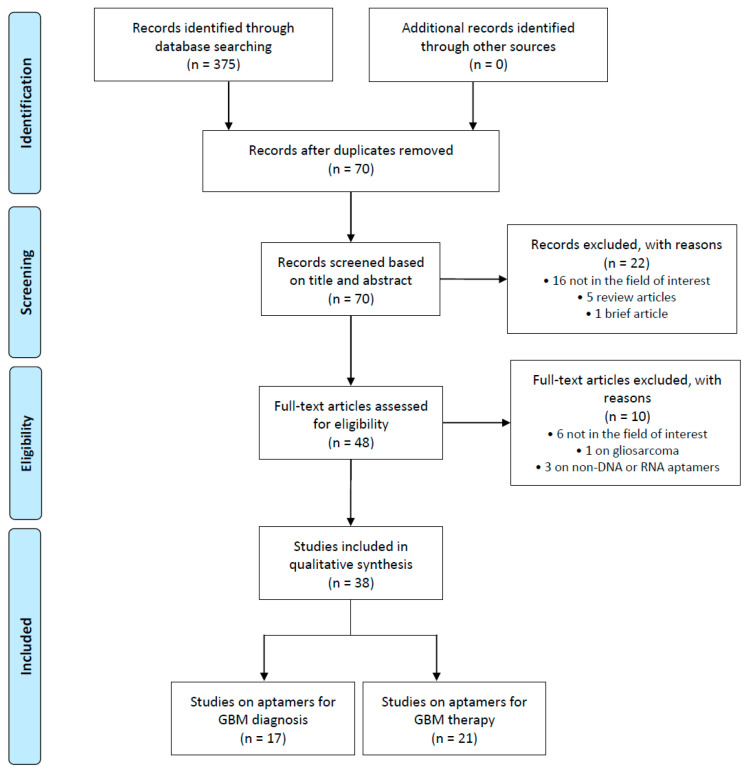
Preferred Reporting Items for Systematic Reviews and Meta-Analyses (PRISMA) flow diagram.

A total of 375 articles was retrieved from the PubMed, Google Scholar, Web of Science, and Science Direct databases. After the removal of 305 duplicate articles, we performed a screening based on titles and abstracts of the remaining 70 articles. Twenty-two records in this step were excluded for the following reasons: 5 were review articles, 1 was a brief article, and the remaining 16 were off-topic and only mentioned the words “aptamers” and/or “GBM” in the abstract. Screening by titles and abstracts yielded 49 potentially eligible articles, which were evaluated by their full text. Of these articles, 1 record was excluded because it involved gliosarcoma, 3 were excluded because they investigated non-DNA or RNA aptamers, and 6 were excluded because they were off-topic and did not investigate the role of aptamers in GBM diagnosis or treatment. Finally, 38 records were included for qualitative synthesis. The PRISMA flow diagram of included studies according to the inclusion and exclusion criteria is reported in Figure 1, and their characteristics are summarized in Table 1 and Table 2.

### 3.2. Aptamers in GBM Diagnosis

Seventeen articles investigated aptamers in imaging and diagnostic systems for GBM. Their characteristics are summarized in Table 1. According to the targets, we distinguish two groups: the first aimed at visualizing Tenascin-C-positive cells, the second at detecting epidermal growth factor receptor- (and its variant III-) expressing cells. In addition, single articles were found for the following GBM-associated proteins: nucleolin, vascular endothelial growth factor receptor (VEGF), and integrin α5β1; and for the following GBM cell lines: T98G, U118-MG, A172, and GBM-initiating cells (also known as GBM stem cells).

### 3.3. Aptamers as Diagnostic

The first aptamers used in GBM detection tools, and thus potentially applicable for GBM diagnosis, were aimed at identifying GBM cells by means of the extracellular matrix glycoprotein Tenascin-C (TN). As early as 2001, Hicke BJ et al. used TN-expressing U251 cells and purified TN to obtain TTA1, an RNA aptamer (modified with f2′-F-pyrimidine and 2′-OH purine nucleotides), specific to the tumor-associated protein TN. TTA1 proved to bind TN with a very high affinity (Kd, 3 × 10^−9^ M) [36]. Moreover, the group evaluated the binding via surface plasmon resonance (SPR), a technique detecting circulating cancer biomarkers usable as key actors of liquid biopsy for GBM diagnosis [57]. A few years later, the same group used scintigraphy to assess the distribution in mice of the technetium-99m-labeled aptamer, showing the potential clinical application of radiolabeled TTA1 [34]. In 2003, the same target was used by Gold et al. to select a DNA aptamer, GBI-10, in the U251 cell line. They demonstrate the selectivity of GBI-10 for GBM cells, with the aptamer-TN interaction assessed via enzyme-linked immunosorbent assay (ELISA) and biosensor analysis with SPR [35]. Thereafter, Li Y et al. emphasize the potential diagnostic application of GBI-10 with a molecular recognition force spectroscopy (MR-FS) study investigating dynamic aptamer-target interactions [29].

More recently, interest has been mainly focused on EGFR and on its receptor variant III, EGFRvIII, which is associated with GBM aggressiveness. Over the past few years, DNA and RNA aptamers have been generated and used for EGFR-mediated detection of GBM. In 2010, Iqbal et al. isolated an anti-EGFR RNA aptamer by iterative selection using the purified human protein [33]. The authors proved not only the ability of the aptamer to bind wild-type and mutant EGFR with high affinity (*Kd* = 2.4 nM), but also demonstrated the aptamer’s capacity to detect and capture murine and human GBM cells when it is immobilized on a glass substrate, so it is possible to use the technique for early detection and the monitoring of residual disease [33]. In fact, one year later, the same group designed a flow-through lab-on-chip device that took advantage of the surface-bound aptamer’s affinity for EGFR, the biomarker overexpressed in GBM, to demonstrate that a microfluidic-based approach can be used to detect and isolate GBM cells [31]. The group then advanced the diagnostic use of anti-EGFR aptamers with two subsequent articles on the tracking of the differential dynamics of GBM cell morphology on aptamer-grafted substrates, and on the analysis of dynamic morphology in computational single-cell metrics to detect and recognize tumor cells [22,29].

Working on the same target, Tan et al. and Wu et al. performed two-cell-systematic- evolution- of-ligands-by-exponential-enrichment (SELEX) selections on U87-EGFRvIII cells in order to specifically recognize receptor variant III. Both groups isolated DNA aptamers that proved to be usable in GBM diagnosis. In fact, Tan et al. demonstrated, in vitro, the imaging of different selected FITC-labelled aptamers in U87-mutated cells [26]. Furthermore, the authors measured the binding affinity of the aptamers by biotin-avidin ELISA (BA-ELISA) assay: all the sequences analyzed had a Kd less than 100 nmol/L. By contrast, Wu et al. used imaging with single-photon emission computed tomography (SPECT), in vivo and ex vivo, with tumor-bearing mice [25]. This nuclear medicine imaging technique is similar to scintigraphy, the conventional diagnostic radioisotope-based approach, but has the ability to provide 3D details and, therefore, is useful for solid tumors like GBM.

Despite a greater focus on these two targets, there are many other groups concentrating their forces on detecting GBM by targeting other tumor-related proteins. Indeed, Hadizadeh et al. utilized the ssDNA aptamer AS1411, which targets nucleolin, to generate AS1411-cadmium telluride (CdTe) quantum dots (QDs) [58], which are usable as versatile fluorescent probes and sensors in several applications. The authors prepared CdTe QDs with four different colors through a microwave-assisted reduction method, and then assessed, with a fluorescence microscope, the aptamer-QD conjugates. They demonstrated the potential of the conjugates as nanoprobes for GBM imaging in vitro [24]. By contrast, Kim et al. generated an aptamer-based diagnostic nanoprobe by conjugating an anti-VEGFR2 DNA aptamer with magnetic nanocrystals. In vivo tests in an orthotopic GBM mouse model showed a high magnetic resonance (MR) imaging sensitivity of the tumor vasculature [27]. Moreover, in the last year, Choulier et al. combined protein- and cell-SELEX to isolate RNA aptamers that selectively bind the integrin α5β1, an αβ heterodimeric cell surface receptor associated with tumor angiogenesis and GBM aggressiveness (Kd in the nM range). The authors prove the diagnostic potential of the labeled aptamer, using it in a fluorescence-based assay on cell lines and in histo-fluorescence assays on patient-derived xenografts (PDXs) [20].

Other groups, such as Kang et al. and Bayrac et al., have used aptamers in microscopy imaging. The former identified high affinity ssDNA aptamers for GBM with the use of U118-MG cells: confocal images show cell and human tumor tissue section staining after FAM- or Cy5-labeled aptamers treatment, respectively [30]. The latter selected GBM-targeting ssDNA aptamers with the use of A172 cells and investigated cell detection via streptavidin-PE staining of the biotinylated aptamer [32]. Both groups have not further investigated the aptamers’ targets in subsequent studies. Similarly, in 2018, Yang et al. selected two aptamers targeting T98G cells—WYZ-41a and WYZ-50a—and, without discovering the targets, proved their diagnostic applicability. Indeed, both aptamers detected their target cells in complex mixtures, such as undiluted fetal bovine serum (FBS) or cerebral spinal fluid (CSF), demonstrating the power of aptamers in improving GBM diagnosis through liquid biopsy [21].

Finally, two groups recently selected aptamers against a specific subgroup of GBM cells, brain tumor-initiating cells (TICs) and GBM stem cells (GSCs), responsible for GBM metastasis and relapse. Rich et al. selected a pool of DNA aptamers recognizing TICs with a very low dissociation constant (Kd between 0.12 and 3.75 nM); binding was proved by aptamer-Cy3-based fluorescence [28]. Some years later, Affinito et al., in 2019, similarly selected the GBM-specific 2′-F-RNA aptamer A40s, using an RNA library on primary human GSCs. The authors demonstrated A40s-mediated detection in GBM cells and in human GSCs tissue sections [19]. Here too, the aptamer proved to have a high affinity for target cells in a low nanomolar range (Kd of 41.92 nM). Since stem cells have a role in metastasis and chemoresistance, these aptamers could be applied in the clinical setting for the early detection of new metastatic niches and for the monitoring of GBM treatment.

Thus, many studies have been carried out to take advantage of the unique characteristics of aptamers to improve the quality and sensitivity of conventional imaging techniques and enhance GBM diagnosis.

### 3.4. Aptamers in GBM Therapy

Twenty-one studies investigated the role of DNA and RNA aptamers in targeted therapy of GBM. Their characteristics are summarized in Table 2. Among the selected studies, 11 investigated aptamer-based therapy with binding inhibiting a specific protein target, and 10 investigated the therapeutic effect of aptamer-based conjugates specifically delivering molecules (non-coding RNA, nanoparticles, chemotherapeutics) to tumor cells after ligand recognition.

### 3.5. Aptamers as Inhibitors

Here we review the therapeutic effect of DNA and RNA aptamers developed as promising new drugs for GBM in the field of targeted therapy. Eleven articles on the therapeutic targets specifically recognized by the aptamers were discussed. We found that half of these studies aimed to block EGFR or its mutated form (EGFRVIII), 3 studies aimed to block the Eph receptor family, whereas fewer investigated aptamers recognizing VEGF, platelet-derived growth factor receptor (PDGFR), or osteopontin (OPN) (one paper for each).

Numerous studies were aimed at selecting inhibitors, including aptamers, for EGFR because it is a relevant feature in primary GBM (it is overexpressed in 60% of tumors) and its mutation correlates with poor prognosis and tumor aggressiveness [59,60]. In this regard, several aptamers were selected and characterized and all of them produced a good reduction in cell proliferation in vitro.

In 2012, Wan et al. developed a chip to observe cell behavior in vitro after treatment with a 2′-fluoropyrimidine-containing RNA aptamer against EGFR. They found that the anti-EGFR aptamer reduces migration and proliferation of GBM cells by blocking EGFR phosphorylation [53].

Very recently, Wang et al. selected and characterized an aptamer against EGFR named CL-4RNV616, containing 20-O-Methyl RNA and DNA nucleotides. CL-4RNV616 was tested not only on U87MG glioblastoma cells but also on Huh-7 liver cancer and MDA-MB-231 breast cancer. The authors demonstrated that CL-4RNV616 binds and inhibits EGFR, has high stability in human serum, and increases apoptosis in cancer cells; in breast cancer, biopsy-based immunostaining demonstrated high EGFR, but no data were given on GBM tissues [40].

Other studies selected and characterized aptamers targeting a variant of EGFR named EGFRvIII, which correlates with EGFRwt amplification in clinical GBM samples. EGFRvIII is constitutively active thanks to a particular deletion and is found in 25% of tumors, but instability makes it a difficult target.

Liu et al. described, for the first time, an aptamer for EGFRvIII with a low dissociation constant (Kd). Unluckily, the aptamer selected in *Escherichia coli* for EGFRvIII was not able to bind the human EGFRvIII protein, the reason why the aptamer was not investigated further [56]. However, two promising aptamers for human EGFRvIII are (i) CL4, a 2′-fluoropyrimidine-containing RNA aptamer which binds wild-type and mutant forms of EGFR [50], and (ii) U2, a DNA aptamer specific to the above-mentioned EGFR variant [44].

In vitro, CL4 proved to be a promising inhibitor. In fact, the authors nicely demonstrate that CL4 inhibits GMB growth as well as or better than the current EGFR inhibitor (gefitinib), representing, therefore, an attractive alternative for GBM management. Moreover, solid evidence demonstrates that EGFR inhibitors make the growth and survival of GBM cells dependent on PDGFRβ signaling, the reason why being in order to improve the therapeutic effect of CL4. Camorani et al. combined CL4 with a second aptamer, named Gint4, which binds and inhibits the phosphorylation of PDGFRβ [50]. Gint4 was previously characterized in vitro and in vivo, with the authors demonstrating a drastic inhibition of cell viability and tumor growth [52]. The combined therapy resulted in a stronger reduction of GBM aggressiveness when compared to TMZ or approved PDGFRβ and EGFR inhibitors (imatinib, gefitinib, and cetuximab) [50]. Instead, the DNA aptamer U2 inhibits tumor growth and acts as a molecular imaging probe in vitro and in vivo [56].

Other promising therapeutic targets for GBM are represented by Eph receptors, known to be involved in tumor invasion and metastasis [61]. Within the family, EphA2 is highly expressed in GBM, but it is not detectable in the normal brain [62]. It correlates with neovascularization [63], proliferation, pathological grade, and patient survival [64,65,66]. Moreover, EphA2 expression increases in GBM stem cells (GSCs), a subpopulation of GBM cells resistant to conventional therapies, and being co-expressed with other stem cell markers, such as CD133 and integrin alpha 7, it represents an important molecular feature of GBM stemness. These well-characterized findings make EphA2 a candidate for efficacious targeted therapy for GBM: in fact, Affinito et al. very recently selected and characterized 2′-fluoropyrimidine-containing RNA aptamers (40L and its truncated form, A40s). As a first attempt, the authors described an innovative cell-SELEX approach aimed at identifying RNA aptamers binding primary GSCs. 40L and A40s recognize not only primary GSCs but also human GBM tissues positive for the GBM stemness marker CD133. Interestingly, A40s binds primary GSCs in the nanomolar range (about 42 nM), and the authors demonstrate that A40s can be used to deliver secondary molecules, for example, microRNAs [19]. The same authors in 2020 published a second paper in which A40s is fully characterized in vitro for its therapeutic effect thanks to the discovery of the aptamer’s target, EphA2.

As expected, considering the relationship between EphA2 expression and GBM progression, the inhibition of EphA2 mediated by A40s treatment reduces self-renewal of GSCs and GBM tumor propagation. A40s is stabile in human serum (~24 h) and crosses the blood brain barrier (BBB), reaching the brain in healthy mice after systemic administration [38]. Although the data support that A40s has potential applicability as a therapeutic tool to block the GSC population and, thus, GBM recurrence, the authors neither investigated in-depth A40s affinity for the other members of the Eph family nor the therapeutic effect of the aptamer in vivo.

EphB3 and EphB2 are reported to be overexpressed in GBM cell lines and to have a pro-tumoral role promoting migration and invasion in cancer cells. GL43.T is an RNA aptamer modified with 2′-fluoro pyrimidine (2′-F-Py) that binds EphB3/EphB2. It blocks cell proliferation and antagonizes migration in GBM cells. The binding affinity for the EphB3 receptor is in the high nM range (433.5 nM–136.6 µM), but the authors do not give any information on the affinity of GL43.T for EphB2 or other Eph receptors [49].

Other approaches have used the bispecific aptamer named 4-1BB–OPN. The authors concomitantly targeted OPN and the costimulatory receptors on CD8+ T cells by using two different aptamers, respectively OPN-aptamer and 4-1BB, attached by an RNA linker sequence. 4-1BB–OPN was tested in vivo in mice with established GL261 GBM cells: the authors solidly demonstrated that the treatment drastically increases survival time by 68%, so is a promising approach to potentiate naturally occurring antitumor immunity via tumor targeting [43].

Another promising biomarker for recurrent GBM is the mediator of angiogenesis, VEGF. The alternative splicing of VEGF mRNA produces four principal isoforms (containing 121, 165, 189, and 206 amino acids), among which VEGF165 is the predominant one. Pegaptanib, approved by the US FDA in December 2004 for treatment of age-related macular degeneration (AMD), is an RNA aptamer targeting VEGF165. Considering that Bevacizumab (a monoclonal antibody against VEGF) was approved for GBM treatment, Verhoeff et al. investigated the effect of aptamer-based anti-VEGF treatment in combination with irradiation in an orthotopic mouse model of GBM. Their results demonstrated that the treatment suppresses invasive growth, increasing progression-free survival (PFS) in vivo [55].

### 3.6. Aptamers as Carriers of Therapeutics

Here, we fully investigate aptamers as main components of targeted delivery systems in GBM. The ten selected articles can be divided into two major groups: those on aptamers as carriers of nanoparticles (five articles) and those on aptamers as carriers of ncRNAs (four articles). Only one article concerned aptamers as carriers of chemotherapeutic agents.

Regarding the first group, Gao et al. published in 2012 the first studies on aptamers as carriers of nanoparticles in GBM. The authors describe ApNP, a conjugate obtained from nanoparticles of MPEG-PCL functionalized with GMT8, a DNA aptamer binding GBM cells. In order to induce GBM cell death, Docetaxel (DTX), an inhibitor of microtubule depolymerization causing mitotic arrest, was used alone as a control or loaded in ApNP (DTX-loaded ApNP). The results show that DTX-ApNP not only induces cell apoptosis and inhibits tumor spheroid growth in vitro, but that it targets GBM tumor mass, prolonging the survival of GBM-bearing mice compared to controls [54].

GMT8 was also tested in association with the PDGFRβ-binding RNA aptamer Gint4.T to deliver tFNA, a three-dimensional (3D) tetrahedral framework nucleic acid to GBM cells. tFNA is a self-assembly of four single-stranded DNAs (ssDNAs) named S1–S4 and represents a promising targeted approach for the delivery of oligonucleotides or drugs. Firstly, the nanoconjugate, named GTG, was characterized in vitro as a delivery tool in two different GBM cell lines (U87MG and bEnd.3). The authors nicely demonstrated that GTG is efficiently internalized in GBM cells and is able to cross the BBB in an in vitro model [41]. When GTG was loaded with paclitaxel (PTX), named GPC-PTX, to investigate anti-GBM efficacy, they found a reduction in cell proliferation and apoptosis.

Fu et al. used the same approach with tFNA functionalized with aptamers for the specific delivery of therapeutic agents in order to kill GBM cells. In this case, tFNA nanoparticles were loaded with TMZ. To confer GBM tissue specificity, ssDNAs named S2 and S3 were elongated with AS1411 and GS24 aptamers, respectively. AS1411, which binds nucleolin and induces tumor cell apoptosis, and GS24, which binds the transferrin receptor (TRF), helped to pass the BBB. The authors demonstrated in vitro that the lethality of tFNA-TMZ was higher than TMZ alone. Furthermore, thanks to GS24, tFNA crossed the BBB in vivo and stayed in brain vessels for 1 h, suggesting that tFNA might be a favorable delivery vehicle [39].

The application of AS1411 as a nanoparticle delivery tool was previously investigated by Luo et al. In this case, poly (L-γ-glutamyl-glutamine) loaded with paclitaxel (PGG-PTX) was functionalized with AS1411. The nanoconjugate exhibited cytotoxicity in vitro on 2D cultures and 3D tumor spheroids. The authors also studied tissue distribution and therapeutic effects of AS1411-PGG-PTX in vivo. They proved that the conjugate’s tissue distribution was related to tumor localization and that the median survival of mice treated with AS1411-PGG-PTX was higher compared to controls [47].

Peng et al. assessed the proprieties of U2-AuNP, a novel brain-targeting complex. As previously reported, the aptamer U2 binds EGFRVIII. U2, thanks to a thiol group, was conjugated to a gold nanoparticle (AuNPs) through an Au-S bond. U2-AuNP inhibited the activation of EGFRvIII, affected U87-EGFRvIII cell proliferation and invasion, and increased the survival of GBM-bearing mice [37].

In the second group of articles, aptamers are used as carriers of ncRNA (miRNA, anti-miR, siRNA). In the first published study, investigating the delivery of small interfering RNAs (siRNAs), the authors characterized the DNA aptamer 32 as a carrier for a c-Met siRNA binding EGFRvIII. Aptamer 32, as well as the c-Met siRNA, were biotinylated (Ba), and both moieties incubated with streptavidin containing four binding sites to form a stable conjugate. The conjugate was characterized in vitro, demonstrating that the Ba aptamer is an efficient delivery tool for c-Met siRNA into U87-EGFRvIII cells, offering a combined treatment regimen for patients with high expression of c-Met and EGFRvIII [44].

The next two studies investigated aptamer-mediated delivery of siRNA targeting STAT3, the role of which as a key regulator in GBM has been demonstrated. In the first, the STAT3 siRNA was conjugated to PDR3, an anti-PDGFRα RNA aptamer. The conjugate strengthens the effect of the aptamer alone: in fact, the authors firstly demonstrated the effect of PDR3 on apoptosis and then confirmed that PDR3-siSTAT3 mediates strong reduction in cell viability in vitro [42]. In the second, Esposito et al. characterized a modified 2′F-Py nuclease-resistant RNA conjugate in vitro and in vivo. In that study, Gint4.T (an anti-PDGFRβ) was conjugated via a sticky bridge approach to ansiRNA specifically targeting STAT3. The conjugate reduced STAT3 mRNA levels and, accordingly, in vitro viability and migration of GBM cells. Gint4.T AsiC proved to lead to a selective functional effect expressed only by PDGFRβ-positive cells. Furthermore, systemic administration of the conjugate in vivo led to reduction of tumor mass and inhibition of neovascularization [45].

The same group designed two different conjugates to deliver miRNA/antimiRNA to glioblastoma stem-like cells simultaneously. In particular, GL21.T (an anti-AXL aptamer) was conjugated to miR-137, and Gint4.T (an anti-PDGFRβ aptamer) to anti-miR-10b. By combining the therapeutic potential of the miRNA-based approach and RTKs, the authors found a drastic inhibition of self-renewal and migration GSCs [48]. The other approach delivered a chemotherapeutic agent via a specific aptamer: the ssDNA aptamer GMT-3 was conjugated to the anticarcinogenic drug Doxorubicin (DOX), known to intercalate into DNA strands by noncovalent interaction. The authors demonstrated in GBM cells that GMT-3 delivers DOX well to GBM cell lines, with a selective release of the cytotoxic drug in targeted cells, overcoming the cytotoxic effects of the chemotherapeutic on normal cells [46].

## 4. Discussion

In this systematic review, we highlight the potential value of the RNA and DNA aptamers in the management of GBM. Aptamers have been shown to be useful tools in diagnostic and therapeutic approaches in several diseases [67,68]; an important milestone was achieved in December 2004, when the US FDA approved the first RNA aptamer for the treatment of neovascular age-related macular degeneration (AMD), named Pegaptanib (Macugen).

To date several studies have investigated the diagnostic role and clinical value of aptamers in GBM, a pathology in which the standard approaches of diagnosis and treatment are still limited [69].

In the literature, there are many data collected on aptamers in GBM. One promising clinical trial started in 2019, which uses a pegylated structured L-oligoribonucleotide aptamer (Spiegelmer) that binds and neutralizes CXCL12, named Olaptesed Pegol (NOX-A12) (NCT04121455). Nevertheless, the benefits of using aptamers as diagnostic and therapeutic agents remains unclear. We have carried out a systematic review examining all studies on RNA and DNA aptamers found to be promising as diagnostic and/or therapeutic molecules published since 2000. This qualitative analysis has involved 38 studies: the results and conclusions of the selected studies reveal the great potential of aptamers in the management of GBM. Despite this, the actual use of aptamers is generally still in its infancy.

In particular, there is a more promising group of aptamers: those described for the development of new treatments for GBM; the clinical usefulness of the other categories of aptamers remains to be clarified since they have been only poorly characterized. With regard to GBM treatment, the best-characterized aptamers inhibiting cancer cell proliferation and with good affinity for their targets are Gint4 and U2. The former binds PDGFRβ and has been studied by different groups. Selected against GBM cells, it was initially studied for its great potential as an inhibitor of cell proliferation. It was then further characterized (i) as a carrier in combination with a second aptamer to increase the therapeutic effect on GBM cells, and (ii) as a carrier of ncRNA (siRNA, antimiR) and PTX-loaded tFNA. In addition, Gint4 binds and functions as a carrier against GSCs, suggesting a potential use in combination with chemotherapeutic agents. Very recently, Cerchia et al. published a short communication in which they demonstrated the ability of GINT4 to cross the BBB in orthotropic GBM mice, confirming its great potential for new therapeutic approaches in GBM. The latter binds EGFRVIII and can be used as a therapeutic nanoparticle carrier and as a diagnostic probe, making it a promising tool in GBM management.

Compared to the above, there are fewer studies on other aptamers in the literature, so it was even more complicated to evaluate the ability of those molecules to help in the management of GBM. One way to assess the value of each individual aptamer may be to look for the year of discovery. In fact, it should be noted that some aptamers, listed in Table 1, were discovered some years ago and since then have not been studied further. It can be assumed that they have been abandoned. However, other aptamers, such as A40s, were first published only last year, so it is likely that further studies are underway, and their value will be assessed in the coming years.

Currently, research in the advancement of GBM therapy is focused on tumor-targeted administration. To date, several aptamers with interesting in vitro and in vivo results have been characterized as carriers of a therapeutic load. Among this group, AS1411 seems to be a promising molecule [57]. AS1411 is a DNA aptamer targeting nucleolin, and is now in clinical trials on advanced solid tumors and acute myeloid leukemia (NCT00512083 phase II, completed; NCT00881244 phase I, completed). Other aptamers, such as GMT8, are good candidates for targeted administration approaches because they have great binding capacity and specificity for GBM, and should be further investigated to determine targets.

In parallel, in the diagnostic technology landscape, aptamer immobilization to develop microfluidic devices and dynamic morphology studies using anti-EGFR aptamers seem to be very promising and practically applicable for GBM diagnosis [21,31]. Concerning the possible aptamer manipulations in this field, the most attractive modifications involve aptamer-isotope conjugations, which have demonstrated a great potential the molecular imaging laboratory, like MRI and SPECT. These diagnostic techniques have been demonstrated to be improved with the use of aptamers.

In this scenario, it would be desirable to reach a common view on the use of aptamers in standard GBM diagnosis and therapy. With our systematic review, we have summarized the available data, highlighting the high value of these molecules. However, despite the interesting characteristics of aptamers, several challenges, either intrinsic to the nature of these molecules (RNA, DNA) or linked to GBM, still need to be overcome. While the limits of aptamers are mostly related to their sensitivity to body-fluid nucleases and CpG toxicity, intrinsic limits in the management of GBM are related to the BBB, which limits the number of drugs that can reach the tumor. Future studies should be aimed at improving the stability of aptamers, discovering aptamers that cross the BBB, and testing aptamers in animal models for preclinical testing.

## 5. Conclusions

Although the potential role of aptamers in the management of GBM is only just emerging, giving a clear direction to the development of promising aptamers for the clinic is an important objective. In this regard, our review first describes the DNA and RNA aptamers that have been studied as diagnostic and therapeutic tools for GBM, and then highlights the most promising aptamers and the challenges that need to be met for their use in clinical practice.

## Figures and Tables

**Table 1:** Characteristics of included studies on diagnosis applications of aptamers in glioblastoma (GBM). Kd = dissociation constant.

Author	Year	Aptamer	Nucleic Acid	Target	Tested	Kd	Conjugated Molecule	Diagnostic Application
Affinito et al. [19]	2019	A40s	2′-fluoropyrimidine- RNA		In vitro; Ex vivo	41.92 nM	miRNA; anti-miRNA; Cy5, Alexa488	Confocal fluorescence microscope, histochemistry
Fechter et al. [20]	2019	H02	2′-fluoropyrimidine- RNA	Integrin α5β1	In vitro; Ex vivo	72–277.8 nM	Cy5, Alexa564	Confocal fluorescence microscope, SPR
Wu et al. [21]	2018	WYZ-41a WYZ-50a	DNA	A172 cells	In vitro	75.27–168.56 nM	Cy5, FITC	Fluorescence microscope
Hasan et al. [22]	2018	Anti-EGFR	2′-fluoropyrimidine- RNA	EGFRvIII	In vitro			Dynamic morphology
Mahmood et al. [23]	2015	Anti-EGFR	2′-fluoropyrimidine- RNA	EGFRvIII	In vitro			Dynamic morphology
Alibolandi et al. [24]	2014	AS1411	2′-fluoropyrimidine- RNA	Nucleolin	In vitro		CdTe QDs	Fluorescence microscope
Wu et al. [25]	2014	U2, U8, U19, U31	DNA	EGFRvIII	in vivo; Ex vivo	3.37–16.78 nM	^188^RE	SPECT
Tan et al. [26]	2013	32, 41, 43, 47	DNA	EGFRvIII	In vitro	0.62–37.57 nM	FITC, Biotin	Confocal fluorescence microscope, ELISA
Kim B. et al. [27]	2013	Anti-VEGFR2	DNA	VEGFR2	In vivo	0.12 nM	carboxylated magnetic nanocrystal	MRI
Kim Y. et al. [28]	2013	A1, A2, A3, A4, A5	DNA	TICs	In vitro	0.12–3.75 nM	Cy3	Fluorescence microscope
Li et al. [29]	2013	GB-10	DNA	Tenascin-C	in vitro	110 µM	NHS–PEG18–aldehyde	MR-FS
Kang et al. [30]	2012	GM128, GM131	DNA	U118-MG cells	In vitro; Ex vivo	20–37 nM	FAM	Confocal fluorescence microscope
Wan et al. [31]	2011	Anti-EGFR	2′-fluoropyrimidine- RNA	EGFR and EGFRvIII	In vitro			Microfluidic device
Bayrac et al. [32]	2011	GMT3, GMT5, GMT9	DNA	A172 cells	In vitro	75.27–168.56 nM	Biotin	Fluorescence microscope
Wan et al. [33]	2010	Anti-EGFR	2′-fluoropyrimidine- RNA	EGFR and EGFRvIII	In vitro	2.4 nM	FAM	Fluorescence microscopy
Hicke et al. [34]	2006	TTA1	2′-fluoropyrimidine and 2′-OH purine RNA	Tenascin-C	In vivo		Rhodamine-RED, technetium-99m	Fluorescence microscopy, scintigraphy
Daniels et al. [35]	2003	GB-10	DNA	Tenascin-C	In vitro	150 nM	Biotin	ELISA, SPR
Hicke et al. [36]	2001	TTA1	2′-fluoropyrimidine and 2′-OH purine RNA	Tenascin-C	In vivo	3 nM	Phosphorus-32	SPR

**Table 2:** Characteristics of included studies on therapeutic applications of aptamers in GBM. Kd = dissociation constant.

Author	Year	Aptamer	Nucleic Acid	Target	Tested	Kd	Conjugated Molecule	Therapeutic Application
Peng et al. [37]	2020	U2	DNA	EGFRvIII	In vitro; In vivo		Gold nanoparticle (AuNPs)	Growth inhibition in vitro and prolongs the survival time in vivo
Affinito et al. [38]	2020	A40s	2′-fluoropyrimidine- RNA	EphA2	In vitro	0.76 ± 0.2641 nM		Growth inhibition, stemness, and migration of GSCs
Fu et al. [39]	2019	GS24	DNA	Transferrin receptor	In vitro; In vivo		tFNA-TMZ	
Wang et al. [40]	2019	CL-4RNV616	20-OMe/DNA mixmer	EGFR	In vitro	18.24 nM		Growth inhibition and apoptosis induction
Shi et al. [41]	2019	GMT8 and Gint4.T	DNA; 2′-fluoropyrimidine- RNA	Unknown; PDGFRβ	In vitro		tFNA-paclitaxel	Growth inhibition, migration, and invasion and apoptosis induction of GBM cells.
Yoon et al. [42]	2019	PDR3	2′-fluoropyrimidine- RNA	PDGFRα	In vitro	0.25 nM	STAT3 siRNA	Growth inhibition, apoptosis induction.
Wei et al. [43]	2019	4-1BB-OPN	2′-O-methylation for all C and U nucleotides	OPN and 4-1BB	In vivo			Immunostimolatory effect and increases survival rate
Zhang et al. [44]	2018	U2	DNA	EGFRvIII	In vitro; In vivo	6.27 nM	^RE^188	Growth inhibition, radiosensitivity, and radiotherapy
Esposito et al. [45]	2018	Gint4.T	2′-fluoropyrimidine- RNA	PDGFRβ	In vitro; In vivo		STAT3 siRNA	Growth inhibition and migration in vitro and inhibition of tumor growth and angiogenesis in vivo
Bayrac et al. [46]	2018	GMT-3	DNA	A-172 cell line	In vitro		Doxorubicin (DOX)	Cytotoxic effects
Luo et al. [47]	2017	AS1411	DNA	Nucleolin	In vitro; In vivo		poly (l-γ-glutamyl-glutamine)-paclitaxel (PGG-PTX)	Pro-apoptotic effect, increases median survival time and cell apoptosis in vivo
Esposito et al. [48]	2016	GL21.T; Gint4.T	2′-fluoropyrimidine- RNA	Axl; PDGFRβ	In vitro		miR-137; antimiR-10b	Inhibition of GSC propagation
Amero et al. [49]	2016	GL43.T	2′-fluoropyrimidine- RNA	EphB3 and EphB2	In vitro	433.5 nM		Cell migration
Camorani et al. [50]	2015	CL4; Gint4.T	2′-fluoropyrimidine- RNA	EGFRvIII; PDGFRβ	In vitro			Growth migration and invasion inhibition
Zhang et al. [51]	2014	32-biotin (BA)	DNA	EGFRvIII	In vitro		c-METsiRNA	Apoptosis induction and growth inhibition
Camorani et al. [52]	2014	Gint4.T	2′-fluoropyrimidine- RNA	PDGFRβ	In vitro; In vivo	9.6 nM		Growth inhibition in vitro and in vivo.
Wan et al. [53]	2013	anti-EGFR	2′-fluoropyrimidine- RNA	EGFR	In vitro	2.4 nM		Growth and motility inhibition
Gao et al. [54]	2012	GMT8	DNA	A-172 cell line	In vitro; In vivo		docetaxel-loaded ApNP	Apoptosis induction and growth inhibition
Verhoeff et al. [55]	2009	Pegaptanib	2′-O-methyl purine/2′fluoro pyrimidine with two 2′-ribo purines conjugated to 40 kDa PEG, 3′ inverted dT	VEGF	In vivo	200 pM		Decreases tumor blood vessel density
Liu et al. [56]	2009	E21	2′-fluoropyrimidine- RNA	EGFRvIII	In vitro	33 nM		Apoptosis induction (after transfection)

## Supplementary Materials

The following are available online at https://www.mdpi.com/2072-6694/12/8/2173/s1. S1. Key terms used in literature search; S2. PRISMA Checklist.
